# Transportation infrastructure planning in supporting disaster mitigation: Case study in Mount Gamalama

**DOI:** 10.4102/jamba.v14i1.1123

**Published:** 2022-03-31

**Authors:** Efendhi P. Raharjo, Sri Sarjana, Mega Safitri

**Affiliations:** 1 Department of Land Transportation, Faculty of Land Transportation, Politeknik Transportasi Darat Indonesia, Bekasi, Indonesia

**Keywords:** mitigation, disaster-prone, Mount Gamalama, Pacific ring of fire, transportation infrastructure, evacuation, volcanic eruption, disaster-prone zone

## Abstract

Effective mitigation planning is needed for communities living in areas prone to disasters, including natural calamities such as volcanic eruptions. The development of disaster evacuation routes in disaster-prone areas, including the area where this study was conducted, requires proper planning in transportation infrastructure. Ternate city in Indonesia is a disaster-prone area because of the presence of an active volcanic mountain and an area traversed by the Pacific ring of fire. This area includes a vulnerable zone that has the potential for disasters such as volcanic eruptions, therefore it is important to make mitigation plans to assist the community in evacuating and reducing the impacts of a disaster. Data was obtained from road network observations and by carrying out inventory surveys on the condition of facilities and infrastructure for land transportation and sea transportation in Ternate city, which is located close to Mount Gamalama. A quantitative approach was utilised in this study through transportation modelling using Vissim to analyse the existing traffic conditions and forecasting. The research aims to formulate disaster mitigation measures to reduce the damages caused by the volcanic eruption of Mount Gamalama and identify plans for evacuation routes in disaster-prone areas. The road network performance of Ternate city showed that the city has roads that can be used as evacuation routes for disaster victims. It has good road performance in terms of meeting points and final evacuation points. Efforts to reduce the number of victims when a volcanic eruption occurs, the socialisation of disaster mitigation to the community and the installation of disaster information signs need to be equipped with the preparation of evacuation routes in the form of evacuation gathering points and final evacuation points. This study recommends local governments to develop new evacuation routes in disaster-prone areas and increase evacuation capacity.

## Introduction

Indonesia is one of the most disaster-prone countries in the world (Fernalia et al. 2020; Yulianto, Utari & Satyawan 2020). Ternate city is one of the regions in Indonesia which is prone to disasters as it is characterised by the interaction of three major plates of the world, namely Eurasia, Australian Indies, and Pacific, and is passed by the Pacific ring of fire. The Mount Gamalama with an altitude of 1715 m above sea level is one of the active volcanoes that often results in volcanic eruptions, lava flows and volcanic ash emissions. This mountain experienced an increase in volcanic activity in 2011 and thereafter 67 activities have been recorded followed by an eruption. Volcanic eruptions in 2016 caused fatalities and severe damages to residential areas with 35 deaths, 72 severe injuries, 120 moderate injuries, 82 severe house damages and 110 moderate house damages. The main reasons for the increased number of fatalities and damage was the absence of an integrated evacuation route for disaster victims, delays in evacuation process and in providing information to community, and suboptimal utilisation of transportation functions in the evacuation process of disaster victims (Diyanayati 2014; Kitamura et al. 2020).

Ternate city is the gateway to North Maluku, a centre for economic growth and regional development that requires coordinated and integrated planning. Therefore, it is necessary to take strategic steps to protect the residents from the threat of the natural disaster that is caused by the volcanic eruption of Mount Gamalama. Ternate city regulation number 2 of 2012, concerning the spatial plan of Ternate city in 2012–2032 (2012), mentions the strategies needed to develop disaster evacuation routes in disaster prone areas. These include improving the quality and coverage of transportation, telecommunication, electricity and water resources which should be integrated and evenly distributed on the islands in Ternate region. Therefore, it is necessary to study the planning of evacuation routes for the disaster victims of a possible volcanic eruption of Mount Gamalama by determining evacuation points and integrated rescue routes for the evacuation of victims and by knowing how to better utilise transportation facilities and infrastructures. The purpose of this study is to guide the formulation of disaster mitigation measures in the event of a volcanic eruption of Mount Gamalama as an effort to reduce the impact of the disaster and plan evacuation routes in disaster-prone areas.

## Literature review

The mitigation paradigm (Tao & Run-qiu 2017), which was previously responsive, is now being transformed into preventive activity so that risks can be minimised (Faturahman 2019). The early warning system is a process of reducing the impact of risks and hazards for volcanic disaster management which is described in the mitigation and preparedness stages (Dewanti, Ayuwat & Yongvanit 2019b). The stages of disaster management used include prevention, preparedness, relief, and reconstruction, but in practice they are only used for warning and monitoring (Van Westen 2000).

The speed of reconstruction carried out on transportation infrastructure has an important impact in increasing the effectiveness of social and economic recovery for communities that have suffered damage caused by disasters. Thus, resource management has a key role in creating an effective reconstruction of transportation infrastructure (Rouhanizadeh & Kermanshachi 2020). In disaster conditions, transportation infrastructure must be able to ensure an efficient, fair, and resilient system (Morshed et al. 2021). Reconstruction of transportation infrastructure aims to identify factors that contribute to disaster mitigation in order to predict construction costs (Safapour & Kermanshachi 2021). All transportation infrastructure needs are not necessarily recoverable in terms of costs because they have uncertain capacity (Itoh 2018).

## Volcanic disaster management

When a volcanic disaster occurs, vulnerable communities with low abilities may find it especially difficult to deal with the situation, therefore there is a need for volcanic disaster management to reduce casualties and property losses (Listyaningsih 2010). Volcanic disaster management map is needed to work out evacuation plans and other possible disaster management strategies (Anyoji 2013). Volcanic eruption disasters have limited emergency planning and mitigation, so it is necessary to immediately develop risk assessment and decision-making methods in crisis conditions in areas of active explosive volcanism (Baxter et al. 2008). Fatalities and injuries related to volcanic eruptions can be minimised by making disaster vulnerability maps in vulnerable areas (Maulana, Prasetyo & Wijaya 2017). The risk of mountain disasters can be reduced by involving the participation of local communities directly through the development of an environmental communication system that needs to be developed for an early warning system (Kusumayudha, Lestari & Paripurno 2018). The early warning system is one of the most important phases in disaster management which aims to reduce the risk during an eruption (Dewanti et al. 2019b). Preparedness is an important effort that is needed to reduce the impact of natural disasters and it is the responsibility of government and society and should reach all levels of society without exception (Fernalia et al. 2020). Because of the high intensity and complexity of disasters, systematic efforts should be made for disaster management (Saputri, Imaniar & Putri 2019). The integration of information systems for early warning of disaster is needed to increase the speed of information, which includes the utilisation of the digital weberianism bureaucracy (Meilani & Hardjosoekarto 2020). It is necessary to estimate the potential for large-scale rock volume and mass impacts from volcanic events that can be applied through tephra transport models that take advantage of varying wind parameters (Asano & Nagayama 2021).

## Disaster victim mitigation and evacuation

Mitigation means taking actions to reduce the effects of a disaster (Gosal, Tarore & Karongkong 2018). It includes a series of activities that are taken by an authorised institution to provide immediate warning to the public about the possibility of a disaster occurring in a place to reduce disaster risk for people living in disaster-prone areas (Maulana et al. 2017). Disaster mitigation is the first step in disaster management that aims to reduce disaster risks and greater impacts (Faturahman 2019). The purpose of mitigation is to reduce the possibility of risk, reduce the consequences of risk, avoid risk, accept the impact of risk, and disseminate risk information (Kusumasari 2014). Modelling impacts in volcanic eruption mitigation scenarios and consequences for disaster prone areas requires realistic efforts for disaster planning and demonstrating the potential risks or benefits of mitigation actions through timely evacuation efforts, consideration of protecting buildings, infrastructure and hardened lifelines (Baxter et al. 2008).

The ability of communities to evacuate contributes to reducing risk (Jumadi, Carver & Quincey 2016). The term ‘evacuation’ refers to the movement of people from their place of residence because of security disturbances or population displacement as a result of natural disasters. Therefore, it is necessary to choose the right evacuation route and an evacuation location that will help reduce losses and save human lives (Atmodjo, Sangkawati & Setiaji 2015). Infrastructure inequalities affect policies in the implementation of transportation safety strategies, especially in the evacuation process during volcanic eruptions (Barajas 2021). The evacuation of people on large scale is a very complex task and depends on efficient use of transport system, and effective evacuation schemes (Sahroli & Hardiansyah 2019). Temporary evacuation sites are generally buildings that do not get enough attention by local government which makes them look dirty and unkempt (Lakosa & Alhadi 2019). The level of vulnerability needs to be understood as a factor that will predict the potential for disasters that will occur (Gosal et al. 2018). The level of disaster risk is determined by the potential for disasters, mitigation efforts and preparedness in dealing with disasters (Pitang, Irman & Nelista 2019). The disaster management capacity of households living in disaster-prone areas can be increased by improving network access (Dewanti, Ayuwat & Yongvanit 2019a). Contemporary issues of emergency planning and risk management need to be developed to deal with worst-case scenarios and the vulnerability of the community that can be learned from the eruption of Mount Vesuvius (Martin 2020). Changes in land use due to volcanic eruptions can have an impact on the modification of transportation networks that encourage more effective mitigation in line with regional development (Cardwell et al. 2021).

## Methodology

To reduce the number of disaster victims and increase public knowledge to be more alert in dealing with disasters, it is necessary to plan the evacuation route for the possible victims. Primary data is obtained from land transportation infrastructure and facilities inventory surveys, marine transportation facilities and infrastructure inventory surveys, and road network observations. Meanwhile, secondary data is obtained from Disaster-Prone Zone (KRB) maps, road network maps and maps of the study area, which are obtained from the public works department and regional disaster management agencies in Ternate city.

Data processing used origin-destination (O/D) matrix, road network maps, as well as transportation facilities and infrastructure. A quantitative approach was used in this study through transportation modelling. The Vissim is a transportation modelling programme that can analyse existing traffic conditions, and forecast. Vissim is a software for microscopic traffic simulation (Yulianto & Munawar 2017). It is able to present simulations with various types and characteristics of vehicles using several alternative points, with the use of transportation modes, determination of evacuation routes, selection of the most effective evacuation routes, installation of information signs, and outreach to the public. Data that has been collected is then codified, structured, and shaped according to predetermined format. Ternate city consists of North Ternate which includes 14 sub-districts, Central Ternate that includes 15 sub-districts, South Ternate that cover 17 sub-districts and Ternate island which cover 13 sub-districts. Secondary data is a summary of data to support analysis process, for example a map of the road network, population, type of crater, and wind direction.

### Ethical considerations

This article followed all ethical standards for research without direct contact with human or animal subjects.

## Result and discussions

**FIGURE 1:**
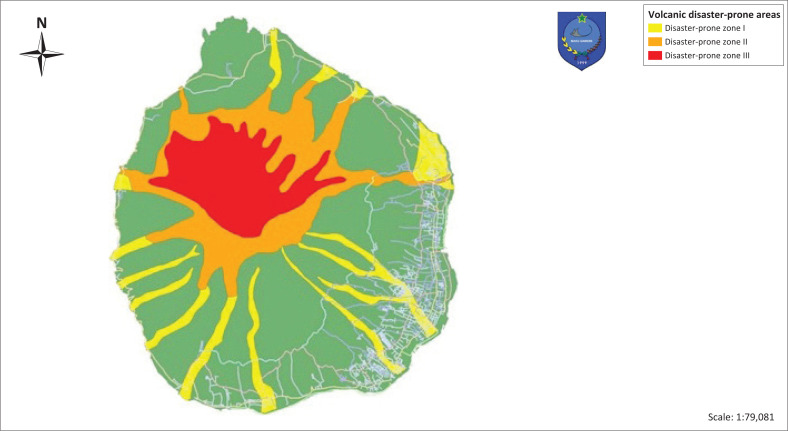
Disaster-prone zone map in Mount Gamalama.

The determination of the volcanic eruption hazard index is made by referring to guidelines issued by volcanology and geological disaster mitigation centre, using the method of weighing the KRB (Yuniartanti 2021). Each KRB in zone I, II, and III consists of flow zone and a fall zone which is given different weight values based on the level of vulnerability (see Figure 1). Another danger caused by increased volcanic activity is the hazards of cold lava flow. Hot cloud is a mass flow of gas and ash during volcanic eruptions. Hot clouds glide at a speed of about 90 km/h with a temperature of about 350°C with a glide distance of 8 km – 12 km with the glide direction following the flow of a valley or river. Apart from the heat that can burn anything in its path, its mass also has ability to destroy large objects. The mass of clouds places volcanoes in their own type of eruption. Volcano experts classify the types of eruptions to anticipate dangers that can be caused. Volcanic hot clouds can result from avalanches or falling lava domes or eruptions. The landslide that occurred in part of the incandescent lava dome caused magma to push on the dome that was being built, causing an avalanche of hot clouds. During the eruption process it can cause lava that is thick enough to cool suddenly. The high level of fragmentation characterised by the spread of tephra over a large area of gas, thick ash and pumice is characteristic of the eruption. Volcanic ash usually leads to residential areas in the Central Ternate District and South Ternate District.

**FIGURE 2:**
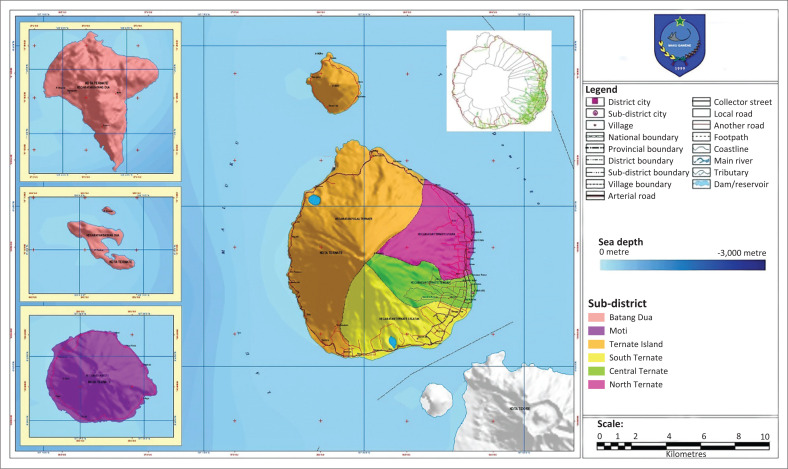
Map of road network and zone of Ternate city.

Ternate city is an archipelagic city whose territory is surrounded by the sea (Hasan 2021). It has an overall road length of 363.9 km, comprising 44.13 km of long provincial roads and 319.77 km of long city roads. The Ternate area is dominated by roads with type 2/2 that are not divided, with intersection types without signals and priority. Ternate city has a combination of grid and radial road network patterns, where as the whole city has radial or circular road network pattern following the contours of land as it surrounds the Ternate islands and at the city centre has grid road network pattern with many intersections. The road grouping according to their status is divided into three categories according to the primary data obtained consisting of 44.13 km long primary collector roads, 20.47 km long secondary collector roads, and 11.49 km long local roads (see Figure 2).

The disaster-prone zone has 3 KRB zones. KRB-I is the area that is exposed to the expansion of hot clouds and lava flows and may be affected by lava flows. This area is also exposed to predicted heavy ash rain with radius of 3.5 km from the centre of eruption. In the KRB-I area, the community needs to be vigilant for the event of eruption of the Mount Gamalama or heavy rain, monitoring any development of the Mount Gamalama activities as forecasted by the Volcanology and Geological Disaster Mitigation Center. KRB-II is the area that has potentials to be affected by hot clouds, ejection and avalanches of incandescent rock and lava flows, especially around the upper slopes of the north, northwest and southwest sides. This area is also potentially threatened by throwing incandescent rocks with a size of 2 cm – 6 cm, as well as heavy ash rain with a radius of 2.5 km from the centre of eruption. KRB-III is the area that has the potential to be affected by hot clouds, ejection and avalanches of incandescent rock and lava flows and toxic gases, especially around peaks and northern slopes. This area has the potential for throwing incandescent stones with a size of more than 6 cm, as well as heavy ash rain with radius of 1.5 km from the centre of eruption. This area is not suitable for building permanent residences and commercial agricultural purposes.

**FIGURE 3:**
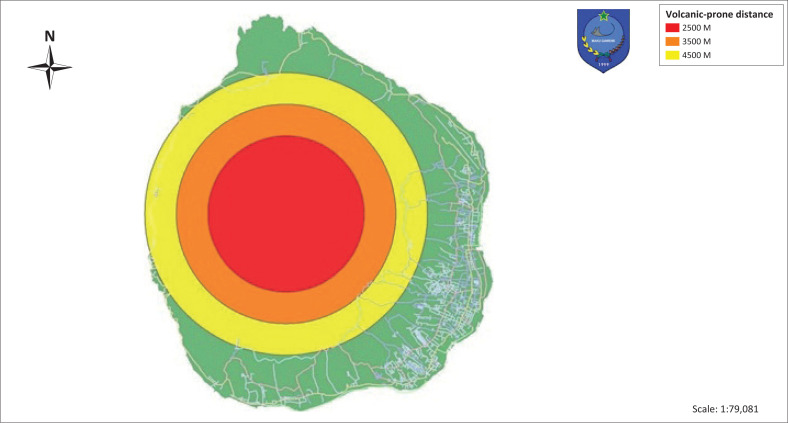
Gamalama mountain prone zone.

**FIGURE 4:**
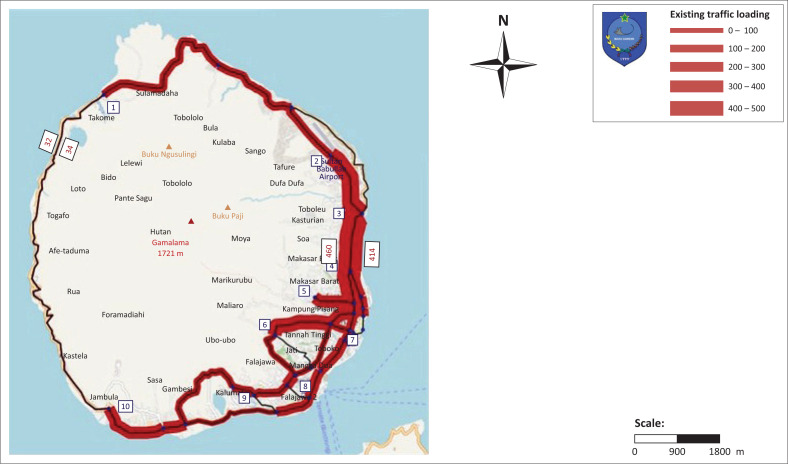
Analysis results on existing traffic loading.

Volcanic areas are depicted with three colours indicating the level of vulnerability (see Figure 3). The red colour on the map indicates the most vulnerable area at a radius of 2500 m, orange colour 3500 m, and yellow 4500 m. The utilisation of the road network used for evacuation routes is shown in the area depicted in yellow. The performance of road network is applied using vissum application to measure the speed of vehicles on the road and the selection of roads used for the selection of evacuation routes. The implementation of road network performance is relevant to regulations in the Regulation of the Minister of Transportation Number 96 of 2015 concerning traffic management and engineering that have conformity in service level category C. The service level category C indicates that the traffic flow is in a stable condition and the traffic density level is in moderate category so that driving speed needs to be limited (see Figure 4).

The model validation proves that this model can be used to forecast trips in a planning year. The validation step used the chi-square test which aims to validate the volume of model results with the volume of survey results so that the use of resulting model is determined. The steps for calculating the chi-square statistical test include formulating hypotheses and determining 95% significance level. Chi-square distribution with the number of road samples tested is 48, so that the chi square obtained from table is 48.60237.

The statistical test is used to check whether the resulting simulation results have significant difference value. If there is no significant difference, then simulation results can be accepted, and any further validation is not necessary as the model results are same as survey results. Otherwise, if there is significant difference, then simulation results cannot be accepted. Table 1 shows the results from the selection of traffic routes and function suitability test using chi-square test to find out whether the model can be acceptable or not.

**TABLE 1:** Validation of Ternate city road network model in 2020.

No	Road name	Capacity	Model	Survey	% Validation	Chi-Square	Model	Survey	Differences
			(Volume)	(Volume)	(Test)	(Test)	(V/C)	(V/C)	
1	Jl. Batu Angus	1414	147	149	1	0.03	0.1	0.11	2
2	Jl. Batu Angus	1414	161	164	2	0.06	0.11	0.12	3
3	Jl. Pemuda	1241	460	463	1	0.02	0.37	0.37	3
4	Jl. Pemuda	1241	414	417	1	0.02	0.33	0.34	3
5	Jl. Merdeka	1608	415	419	1	0.04	0.26	0.26	4
6	Jl. Merdeka	1608	388	391	1	0.02	0.24	0.24	3
7	Jl. Pahlawan Revolusi	1548	201	207	3	0.18	0.13	0.13	6
8	Jl. Pahlawan Revolusi	1548	242	248	2	0.15	0.16	0.16	6
9	Jl. Mononutu	1500	278	281	1	0.03	0.19	0.19	3
10	Jl. Mononutu	1500	227	232	2	0.11	0.15	0.15	5
11	Jl. Jend. Ahmad Yani	856	50	59	15	1.62	0.06	0.07	9
12	Jl. Jend. Ahmad Yani	856	56	60	7	0.29	0.07	0.07	4
13	Jl. Hasan Esa	1382	182	190	4	0.35	0.13	0.14	8
14	Jl. Hasan Esa	1382	166	170	2	0.1	0.12	0.12	4
15	Jl. Raya Mangga Dua	1261	124	129	4	0.2	0.1	0.1	5
16	Jl. Raya Mangga Dua	1261	124	130	5	0.29	0.1	0.1	6
17	Jl. Raya Bastiong	1106	301	308	2	0.16	0.27	0.28	7
18	Jl. Raya Bastiong	1106	274	280	2	0.13	0.25	0.25	6
19	Jl. Kalumata I	1079	230	234	2	0.07	0.21	0.22	4
20	Jl. Kalumata I	1079	237	240	1	0.04	0.22	0.22	3
21	Jl. Poros Ngade I	1079	91	99	8	0.7	0.08	0.09	8
22	Jl. Poros Ngade I	1079	85	89	4	0.19	0.08	0.08	4
23	Jl. Univ. Khairun II	695	133	136	2	0.07	0.19	0.2	3
24	Jl. Univ. Khairun II	695	126	129	2	0.07	0.18	0.19	3
25	Jl. Jambula	1079	168	170	1	0.02	0.16	0.16	2
26	Jl. Jambula	1079	158	160	1	0.03	0.15	0.15	2
27	Jl. Kapitan Patimura	619	212	215	1	0.04	0.34	0.35	3
28	Jl. Kapitan Patimura	619	219	232	6	0.77	0.35	0.37	13
29	Jl. Yos Sudarso I	1482	124	129	4	0.2	0.08	0.09	5
30	Jl. Yos Sudarso I	1482	124	130	5	0.29	0.08	0.09	6
31	Jl. Jati Besar I	619	106	109	3	0.08	0.17	0.18	3
32	Jl. Jati Besar I	619	113	117	3	0.14	0.18	0.19	4
33	Jl. Kalumata II	695	108	115	6	0.45	0.16	0.17	7
34	Jl. Kalumata II	695	119	125	5	0.3	0.17	0.18	6
35	Jl. Univ. Khairun	695	32	37	14	0.78	0.05	0.05	5
36	Jl. Univ. Khairun	695	34	39	13	0.74	0.05	0.06	5
37	Jl. Gambesi – Sasa	695	168	174	3	0.21	0.24	0.25	6
38	Jl. Gambesi – Sasa	695	158	163	3	0.16	0.23	0.23	5
39	Jl. Stadion	1032	241	244	1	0.04	0.23	0.24	3
40	Jl. Stadion	1032	240	245	2	0.1	0.23	0.24	5
41	Jl. Kampung Pisang	695	157	160	2	0.06	0.23	0.23	3
42	Jl. Kampung Pisang	695	136	139	2	0.07	0.2	0.2	3
43	Jl. Facei – Tarau	1079	329	332	1	0.03	0.3	0.31	3
44	Jl. Facei – Tarau	1079	313	315	1	0.01	0.29	0.29	2
45	Jl. Pelabuhan Ferry Bastiong	1044	182	188	3	0.2	0.17	0.18	6
46	Jl. Pelabuhan Ferry Bastiong	1044	166	169	2	0.05	0.16	0.16	3
47	Jl. Univ. Khairun I	695	153	158	3	0.16	0.22	0.23	5
48	Jl. Univ. Khairun I	695	140	143	2	0.06	0.2	0.21	3

A meeting point is a pre-decided designated location where people are expected to be gathered in a disaster incident. Meeting points are needed during the evacuation process with several criteria that must be met, including the availability of adequate open areas, having easy access by disaster victims and volunteer teams, having adequate protection from direct or indirect hazards from disasters, providing temporary places for vulnerable groups, having easy access to mobilise to the location in a relatively short time, availability of first aid facilities, availability of adequate transportation access to get to a safer place quickly, and availability of evacuation route maps that are easy to read and understand quickly. Based on the data from regional disaster management agencies, there are several locations which can be used as evacuation points and final evacuation sites for the victims of a possible volcanic disaster in Mount Gamalama.

The provision of evacuation modes is useful as an input for finding a safe, closest and fastest evacuation place (see Figure 6). The planning for the provision of transportation modes must consider the type of mode used for evacuation process. The provision of transportation modes in the event of a disaster is required during evacuation by considering the travel time. Understanding wind speed is useful for calculating evacuation speed by considering wind direction carrying volcanic ash. The mode of transportation needs to consider the classification of the road to be traversed. The provision of evacuation modes is adjusted to the needs of type for transportation mode and road class.

**FIGURE 5:**
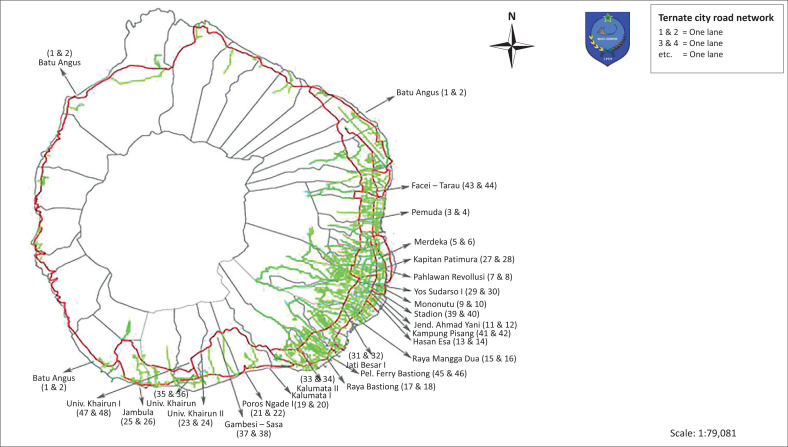
Ternate city road network.

**FIGURE 6:**
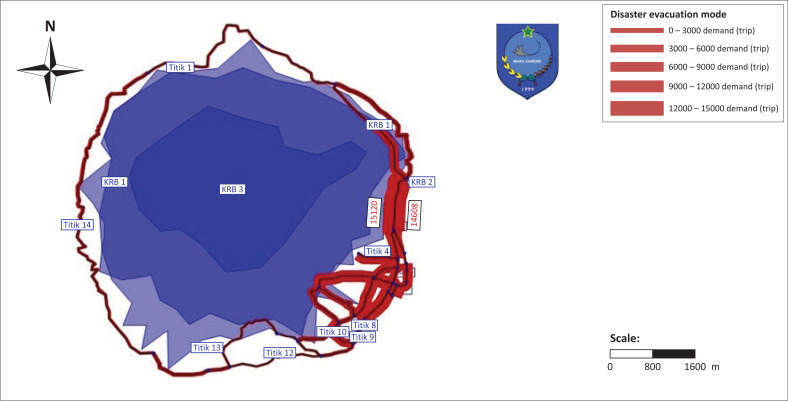
The map of disaster evacuation mode.

It is assumed that all disaster victims do not have private vehicles and the evacuation process is carried out only through existing road access (see Table 2), so that the following formula can be applied:


$$\text{Fleet provider}=\frac{\text{Population }(\text{demand})}{\text{Vehicle capacity}}$$ [Eqn 1]

**TABLE 2:** Fleet requirements.

Meeting point zone	Total population	Resident in coverage area	Own vehicle		Private vehicle	Demand	Evacuation fleet	Health fleet
			Car	Motorcycle	Users			
1	16 892	2567	256	5354	12 244	2081	52	5
2	53 341	5431	978	20 021	45 910	2000	50	6
3	61 839	7232	1023	22 908	51 954	2653	66	8
4	9520	2531	272	2112	5856	1133	28	4
5	9538	2521	253	2132	5782	1235	31	5
6	19 059	3241	457	5212	13 166	2652	66	10

**Total**	**-**	**-**	**-**	**-**	**-**	**11 754**	**294**	**38**

**FIGURE 7:**
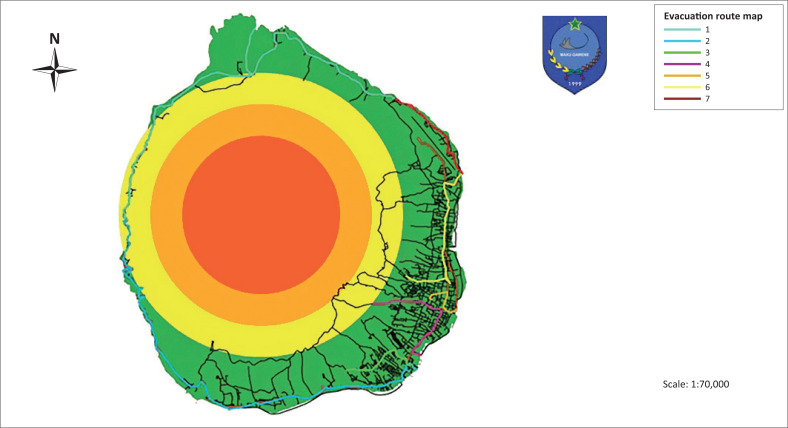
Evacuation route map.

The evacuation route, as well as the meeting points for disaster victims can be decided based on the available test results (Table 2). Figure 7 describes the seven routes used as evacuation routes for disaster victims. Route 1 is meeting point 14 which is on Jikomalamo beach bridges, meeting point 1 at SMP Taduma with final evacuation location at Jikomalamo port which can be accessed via Batu Angus 1 streets, and to Jikomalamo beach streets. Route 2 is implemented as meeting points 13 and 12, where meeting point 13 is located at Gudang Dolog and meeting point 12 is located at Gambesi Hajj hostel, and those used as final evacuation points are at the ferry Bastiong Port and speed boat. The final evacuation point can be accessed via Jalan Jambula to Jalan Poros Ngade, to Jalan Bastiong, to Jalan Bastiong Ferry Port and to Jalan Pasar Bastiong Inpress. Route 3 is meeting point 10 which is located at SD Ubo-ubo and meeting point 11 which is in Kayu-Merah field with final evacuation point being at Bastiong ferry port and speed boat Bastiong, which can be accessed via Kalumata-2 road to ferry port Bastiong street, and to Bastiong inpress market streets. Route 4 is meeting point 8 located in Perikanan field and meeting point 9 is at Bastiong terminal with final evacuation point being at Mangga-Dua port, which can be accessed via inpress Bastiong market street to Semut Mangga Dua port. Route 5 is meeting point 6 located at stadium and meeting point 7 is at Ahmad Yani port, with final evacuation point being at Ahmad Yani port, which can be accessed via Kampung Pisang street to Kapitan Pattimura street, heading to Pahlawan Revolusi street, heading to Ahmad Yani street. Route 6 is planned as meeting point 4 which is located at Kompi-Senapan and meeting point 3 is located in Ngaralamo field with the location of final evacuation point at Dufa-dufa port, which can be accessed via Kapitan Pattimura street section leading to Merdeka street, heading to Pemuda street, and head towards Batu Angus street. Meanwhile, Route 7 is meeting point 2 located at Dufa-dufa port, with the location of final evacuation point at Dufa-dufa port, which can be accessed via Facei Tarau street leading to Batu Angus street.

The community needs to understand the time interval for evacuating themselves when the volcano erupts by following the evacuation pattern and process to go to a safe place within a span of about 5–10 min. The time interval for evacuating victims from the disaster point to the evacuation point is called the golden time. The time needed to survive a disaster is only about 15 min so that the movement of people from their place of origin to their destination must be taken in less than 15 min. Evacuation time is at least 30 to 90 min to get to the final evacuation site. The speed of the vehicle during the evacuation process is an important factor to consider because there will be many vehicles accessing the site. This is because the evacuation time lag from the gathering point to the final evacuation point has very limited time duration. Evacuation is complemented by the notification of which areas are affected by the eruption.

To prevent and minimise potential impacts because of the future eruptions, it is necessary to plan mitigation programmes and disaster preparedness. Mitigation for the community in Ternate City is needed to minimise the risk of disaster when a volcanic eruption occurs. The preparedness of residents for potential volcanic eruptions has an impact on the rate of recovery from the conditions that occur. The Ternate City Government is obliged to strengthen the level of community preparedness in order to mitigate the effects of a disaster. In addition, a spatial plan is needed that is able to anticipate if a volcanic eruption occurs which includes residential areas along the west to south and east coasts of Ternate Island because it has a large population.

The impact of traumatic disasters on people and environment because of volcanic eruptions and earthquakes can be devastating, as they not only result in huge casualties and material and environmental damages, but also drain the economic resources devoted to improving the welfare of a community. Therefore, preventing and minimising impacts through mitigation and preparedness programmes is imperative for the survival and welfare of people living in disaster-prone areas such as Ternate city. The initial step to develop an effective and efficient disaster preparedness and mitigation process in the city of Ternate is to conduct disaster mitigation policy analysis, preparedness surveys and infrastructure assessments.

Volcanic eruption disaster management efforts require monitoring and observing volcanic activity, providing maps of disaster-prone areas, providing information on volcanoes, and improving the quality of human resources. Prevention efforts when an eruption occurs require early warning, disaster management procedures and evacuation of victims to safe locations. Meanwhile, the efforts that need to be made after a disaster has occurred include maintaining an inventory of data related to the distribution and volume of eruptions, identifying risk areas, and repairing the affected facilities.

Disaster signs are information that is placed or installed in disaster-prone areas, in the form of symbols, letters, numbers, sentences, or a combination thereof that serves to provide instructions and warnings for everyone who is in disaster-prone areas. The completeness of signs needed in disaster conditions can be in the form of signposts and signs for attaching signs. Disaster warning signs are needed to provide information and directions for people living in disaster-prone areas. Meanwhile, disaster warning signs are used to inform warnings and threats of disasters or dangerous locations in disaster-prone areas. The disaster information board contains information on disaster-prone areas and their hazards, information on disaster events that have occurred and the possibility to occur again, and the location of temporary gathering places or refugee camps.

## Conclusion

Based on the data analysis of Ternate city road network, it can be stated that the road used as an evacuation route for the victims of Mount Gamalama has good road performance. Evacuation route refers to the meeting point of evacuation and final evacuation point. It is recommended that the mode of transportation used in the evacuation process should be one that has a large capacity. Determination of the type of transportation mode is carried out by considering the ease of access to the evacuation site safely and quickly through seven predetermined evacuation routes by taking into account the location where the meeting point is located. Evacuation is a measure to protect human life which should be carried out with adequate planning to get the intended results (Memito et al. 2020). To reduce the number of victims, proper planning of disaster mitigation is needed for the community. Continuous maintenance of Ternate City transportation facilities and infrastructure which are used in the evacuation process must be carried out properly and consistently. Installation of traffic signs in disaster areas to make it easier to get information is needed to help facilitate access to evacuation of victims. Ease of access can be done by building transportation infrastructure (Sarjana et al. 2020), and infrastructure development in the transportation sector can affect regional development (Hiranrithikorn & Pamornmast 2019). The transportation system, network density, and capacity are the main concepts needed to plan logistics, mitigation and adaptation in the face of disaster risk (Chakwizira 2019). Local governments are advised to develop new evacuation routes in disaster-prone areas, in addition to utilising the existing evacuation routes. The process of creating new evacuation routes can be carried out in stages according to a predetermined plan. Evacuation routes must be prepared with good calculations so that people can use them in emergency (Suryani et al. 2020). The purpose of determining evacuation routes is to increase understanding in disaster mitigation and disaster prevention education (Yamamoto & Li 2017).
